# Novel Approaches to the Prevention and Treatment of Rabies

**DOI:** 10.19070/2330-0027-150002

**Published:** 2015-04-07

**Authors:** CW Gnanadurai, CT Huang, D Kumar, Zhen F. Fu

**Affiliations:** 1 Department of Pathology, College of Veterinary Medicine, University of Georgia Athens, USA; 2 State-key Laboratory of Agricultural Microbiology, College of Veterinary Medicine, Huazhong Agricultural University, China

**Keywords:** Rabies Virus, Pathogenesis, Post-Exposure Prophylaxis, Virus Neutralizing Antibodies, Blood-Brain Barrier, Therapy, Treatment

## Abstract

Rabies is a highly lethal disease caused by the neurotropic rabies virus (RABV), and it remains an important public health problem globally. Effective vaccines have been developed for pre- and post-exposure prophylaxis (PEP). PEP is only effective if it is initiated promptly after recognizing exposure. Once neurological symptoms develop, however, it is widely accepted that there is no effective treatment available. Recent studies indicate that the presence of RABV-specific immunity (i.e. Virus neutralizing antibodies, VNA) and the transient enhancement of the BBB permeability are absolutely required for effective virus clearance from the CNS. In principle, it has been shown in mice using various live-attenuated RABVs or recombinant RABVs expressing three copies of the G or expressing chemokine/cytokines, which can induce high levels of VNA in the serum and also capable of transiently enhancing the BBB permeability that it is possible to clear the virus from CNS. Also, it has been demonstrated that, intravenous administration of VNA together with MCP-1 (shown to transiently open up BBB) can clear RABV from the CNS in both immunocompetent and immunocompromised mice, as late as 5 days after lethal challenge. Novel therapeutic approaches aimed at allowing the peripheral VNA to cross the BBB by administration of the VNA in combination with biological or chemical agents that can transiently open up the BBB would be useful to establish an effective therapy for rabies in humans. In this review, we focus on the some of the approaches that can be used to meet the challenges in the field of rabies treatment.

## Introduction

Rabies (Latin, “madness”) is a highly lethal zoonotic disease caused by a neurotropic rabies virus (RABV) of the Lyssavirus genus, in the family of Rhabdoviridae, order Mononegavirales. These are bullet or rod-shaped enveloped viruses with a negative-sense, single-stranded RNA genome [1]. RABV infects a wide range of hosts, including dogs, cats, raccoons, skunks, foxes, coyotes, bats, and human beings [2]. RABV is usually transmitted to humans through a bite from domesticated or wild animals. It invades the central nervous system (CNS) which leads to acute encephalitis and death [2, 3]. It has been estimated that about 70,000 people die from rabies each year, mostly in Africa and Asia [4]. Effective vaccines have been developed for pre-and post-exposure prophylaxis (PEP). Timely administration of PEP can prevent the development of rabies, when individuals are exposed to the virus. The PEP includes through cleansing of the wound, administration of vaccines and equine or human rabies immune globulins (ERIG or HRIG) [5, 6]. However, the PEP is ineffective once neurological signs have appeared. Yet, there are a few reports on human rabies survivors, but no effective/established therapy is available till date. Improvements in the treatment of rabies are often translated from key studies of its pathogenesis in animal models. The purpose of this article is to review the current literature and to highlight the novel approaches attempted to the prevention and treatment of rabies using animal models.

## Rabies: Virus and Disease

### Molecular characteristics of RABV and epidemiology of rabies

RABV has a non-segmented and negative-strand RNA genome and its genetic information is organized in the form of a helical ribonucleoprotein complex (RNP), in which the linear RNA is tightly associated with the viral nucleoprotein. The genome of RABV encodes for only five proteins in the order: nucleoprotein (N), phosphoprotein (P), matrix protein (M), glycoprotein (G), and the large protein (L, also termed RNA-dependent RNA polymerase, RdRp) [1]. The N plays a critical role in viral transcription and replication [7]. The G forms approximately 400 trimeric spikes, which are tightly arranged on the surface of the virions [8]. The G is a major determinant for RABV neuropathogenicity by binding specific receptor(s), entering the nervous system through the endosomal transport pathway [9] via a low-pH-induced membrane fusion process [10]. The N-L-P polymerase complex starts transcription with the production of a short RNA molecule, the leader RNA, that is neither capped nor polyadenylated. Subsequently, mRNAs are produced for N, P, M, G and L. The switch between transcription and replication of genomic RNAs are controlled by the level of N protein [11]. All transcription and replication events take place in the cytoplasm inside a specialized ‘virus factory’, the Negri body [12].

According to the World Health Organization (WHO), approximately 30,000 people die of rabies each year in Asia [13]. It is estimated that more than 3 billion people are exposed to dog rabies in Asia. One Asian dies every 15 minutes among them 15% are likely to be children under 15 years of age. In India, about 15 million people are bitten by dogs every year, it has been reported that annually 25,000–30,000 deaths occur due to rabies, and around 2,500,000 people undergo PEP. Nepal has one of the highest reported per capita rates of human rabies deaths in the world [14]. Rabies is an important public health problem in Bangladesh, where nearly 100,000 people were bitten by dogs in 2009 and 3,000 died of rabies [13]. In Pakistan, it is estimated about 2,500 deaths occur due to rabies and around 70,000 people undergo PEP treatment [13]. Rabies causes at least 24,000 deaths per year in Africa and the highest death rates are reported in poor rural communities and children [13]. Human rabies has been disappearing from many European and American countries, mainly due to the enforced policy of pet vaccination programs.

### Animal reservoirs and human exposure

Although all mammals are susceptible to RABV, only a few species are important as reservoirs for the disease. Dogs remain the most important reservoirs for rabies in the developing countries of Asia and Africa [15]. In the developed nations, dog rabies has been eliminated or controlled through mass vaccination during the past 70 years [16]. However, wildlife rabies becomes a major concern. In North America, rabies is endemic in raccoons, foxes, coyotes and skunks [16] while fox rabies is endemic in Europe [17]. Wolves, jackals, and other wild animal species have also been reported as reservoirs in other regions [18, 19]. Bats are probably the ultimate reservoirs for RABV [16, 20–22]. In the Americas, a number of bat species carry distinct RABV strains [23], whereas in Europe and Australia bats carry rabies-related lyssaviruses [15].

## Rabies vaccines and PEP

### Current human rabies vaccines and PEP

Successful vaccines have been developed for PEP by Louis Pasteur in 1885. The initial vaccine was prepared from the rabid rabbit spinal cord (nerve tissue); subsequently, the vaccine preparation progressed from nerve tissues to cell cultured RABV. The current PEP consists of through cleansing of the wound and timely administration of vaccine and anti-rabies immunoglobulin (RIG) [5, 24]. PEP can prevent the development of rabies in exposed individuals, only when administered immediately after exposure [25]. The aim of administering rabies specific antibodies at the site of exposure is to immediately neutralize the virus and prevent the virus from entering the CNS [26]. Vaccination is then provided to induce the host immune system to combat the virus during the time period between exposure and the onset of clinical signs (incubation period). Current vaccines used for rabies prophylaxis are inactivated RABV, derived from primary cell cultures. Among the available rabies vaccines, WHO regards the human diploid cell vaccine (HDCV) as the gold standard [27]. The purified chick embryo cell vaccine (PCECV) is prepared from a fixed strain of FLURY LEP grown in primary cultures of chicken fibroblasts, which is used worldwide and shown to be equally effective and cheaper than HDCV [28]. Purified Vero cell vaccine (PVRV) is the most recent cell culture rabies vaccine, which is currently available in more than 100 countries in Europe, Asia, Africa and Latin America. It has been widely and routinely used in many countries for both pre- and post-exposure prophylaxis [29, 30]. Currently, equine or human rabies immune globulin (ERIG or HRIG) for PEP is prepared from pooled sera taken from hyper-immunized horses and humans, respectively. However, ERIG can cause serious adverse reactions while HRIG is expensive for patients, particularly in the developing countries. In addition, its availability is limited worldwide [31].

### Rabies vaccines in pets and wildlife animals

Inactivated RABV vaccines are currently used for routine vaccination of pet animals like dogs and cats, however, multiple immunizations have to be carried out to provide sufficient immunity throughout the life of the animals [32]. Oral rabies vaccines (live-attenuated or live-recombinant vaccines) have been successfully developed for wildlife and two of them are commercially available [33, 34]. Vaccinia virus expressing RABV G (VRG) is found to be an effective oral immunogen for raccoons and foxes under laboratory settings and in the field[35, 36]. Although VRG is safe and effective in vaccinated animals, its exposure to humans can induce skin inflammation and systemic vaccine infections[37]. SAG-2, derived from an attenuated Street Alabama Dufferin (SAD) strain, has been successfully used in Europe for oral immunization of foxes [38, 39]. SAG-2 has also been shown to be safe, immunogenic and effective in dogs in field trails[40]. However, the immunogenicity of SAG-2 is low and only low levels of VNA titers are detected in the immunized dogs [41].

### Rabies Pathogenesis

Despite the long history of human rabies, its pathogenic process remains poorly understood. Most of what we know about the disease process is acquired from investigations conducted in experimental animal models. The overall outcome of an exposure to RABV depends in part upon the rabies genotype (different strains and mutants) or variant involved, its pathogenicity (apoptogenicity, neuroinvasiveness), the dose of virus inoculated (severity of exposure), the route as well as the host species and its susceptibility to the particular pathogen together with innate and adaptive immune responses of the host [42]. However, various studies in animal models indicate that the pathogenic wild-type/street RABV and the fixed (laboratory-adapted) RABV evidently behave differently in each step of their life cycle in the host. RABV G is the only surface protein of the virion and capable of inducing virus neutralizing antibodies (VNA) [43]. The G protein plays an important role in rabies pathogenesis [44] by binding to neural receptor such as acetylcholine receptor [45] and neural cell adhesion molecules (NCAM) [46] contributing to the exclusive neurotropism and neuroinvasiveness of RABV [47]. Virus may enter muscles and replicate at the site of inoculation or enter directly into peripheral nerves without prior replication in non-neural tissues [48]. It is believed that once virus particles enter the peripheral nervous system and start to spread to the CNS, a fatal outcome of the disease is inevitable, though there are some reports of rabies survivors. RABV enters motor and/or sensory axons of peripheral nervous system and spreads to the CNS by retrograde fast axonal transport at a rate of approximately 50–200 mm/day [49]. The pathogenic RABV have evolved specific mechanisms to escape early immune system recognition in the periphery via limited replication, minimized G expression [50, 51], suppression of interferon response, anti-apoptotic stimulation, and transportation through neurons only. On the other hand, fixed RABV induces extensive inflammation by activating innate immune responses [51–53], induces apoptosis [54], replicates to higher levels and express high levels of the G protein [55]. However, the mechanism adopted by the fixed RABVs to elicit immune responses and the wild-type RABVs to evade immune system is still not entirely clear. It has been shown that the innate immune responses and inflammation in the CNS is associated with BBB permeability enhancement [56, 57] in mice infected with fixed RABV but not in those infected with street RABV [53, 57, 58]. Current understanding on the striking difference between pathogenic and non-pathogenic rabies biology is summarized in the Table 1.

### Rabies management and therapy

Rabies is traditionally considered a uniformly fatal disease after onset of clinical manifestations [59, 60]. To this date, there is still no effective therapy for those who develop rabies encephalomyelitis. However, there is now increasing evidence that non-lethal infection can occur in experimental animals as well as in humans [61–66]. There are many rabies case reports, but only a few cases of treated patients have been published. Only a few patients with acute illness have been reported to survive [63, 67–71]. The literature documents five human rabies survivors prior to 2004 [63, 67–70]. However, all of them received rabies vaccine before the onset of the clinical symptoms, but none of them received HRIG. High levels of VNA were detected in both serum and CSF, but no RABV or rabies antigen was detected. Though only one had a full recovery without any neurological complication and all the others had partial recoveries and one of them died within four years due to severe complications. It is assumed that the neurological complications may be due to post-vaccination encephalomyelitis, which have been reported as a side effect of neuronal tissue vaccines [72]. Though these five cases received vaccination before the onset of clinical signs, it does not alter the fact that the mortality rate of the disease is 100% once the critical stage of incubation period is reached without any treatment.

However, recently there are few reports on rabies recovery. A 15-year-old girl started developing a series of neurological symptoms one month after a bat bite exposure [66]. VNAs were detected in both serum and CSF upon hospitalization, and subsequently increased over time. She was then treated with “Milwaukee Protocol” which includes induction of therapeutic coma and administration of combination of antiviral agents, and immunotherapies including rabies vaccination, rabies immune globulin, ribavirin, interferon-α and ketamine. After the treatment, she was discharged from the hospital with neurologic deficits[71]. The most recent case was a 15-year-old boy from Brazil, who was attacked by a hematophagous bat and developed symptoms a month later. Prior to onset of symptoms, he received four doses of rabies vaccine and then was treated with therapeutically induced coma and other therapies (“Milwaukee protocol”). However, the patient survived with severe neurological sequelae. Out of the 5 survivors, treated with “Milwaukee Protocol” three of them had anti-RABV antibodies in CSF prior to treatment. However, since then, there have been at least 20 cases in which the main component of “Milwaukee Protocol” have been used and fatal outcomes have resulted [73]. The mechanisms involved in the prevention of lethal rabies using Milwaukee Protocol is not completely understood thereby, it is regarded ineffective and considered scientifically irrational by some rabies experts in treating human rabies [73–77].

However, one of the major findings associated with non-lethal infections is that many of the survivors had VNA in the serum and /or CSF, and high level of total protein in the CSF, which are likely the key to their survivals [64, 65]. Thus, a combination of therapy pertaining to the induction of CSF VNA should be considered for the effective clearance of rabies viruses from the CNS.

### Immune clearance of RABV from the CNS

It has been thought that it is very difficult to clear RABV once it enters into the CNS [59, 78]. This assumption began to change when it was demonstrated in rats that RABV can be cleared from the CNS by intravenous administration of VNA [25]. Also, it has been demonstrated in mice that clearance of RABV from the CNS requires the presence of RABV-specific immunity (i.e., VNA) in the CNS and enhancement of BBB permeability [79]. BBB is composed of tightly packed endothelial cells, astrocytes end-feet and pericytes which selectively exclude most blood-borne substances from entering the brain [80]. It has been shown by Roy et al., that the lethal SHBRV infection can be prevented by opening the BBB [58]. Failure to open the BBB to deliver immune effectors to CNS leads to lethal rabies [57, 81]. These studies indicate the importance of BBB permeability enhancement in RABV clearance from CNS.

### By using experimental autoimmune encephalomyelitis disease model

The presence of BBB presents a huge challenge for effective delivery of therapeutic agents to the CNS. Many potential drugs against neurological diseases, which are effective at the site of action, have failed, due to their inability to cross the BBB to reach the CNS [82]. Understanding the mechanism involved in triggering the BBB permeability changes in mice clearing RABV and mice developing the CNS inflammatory disease in experimental autoimmune encephalomyelitis (EAE) might provide insight into how the therapeutic agents can be delivered across the BBB without neuropathological complications [56]. EAE is an animal model of multiple sclerosis (MS). MS is a chronic autoimmune disease of the CNS characterized by the breakdown of BBB and accumulation of inflammatory infiltrates in the CNS [83, 84]. Many similarities exist between the MS and rabies in terms of CNS immune pathology i.e. inflammation and demyelination[85]. However, in EAE elevated BBB permeability is associated with the development of neurological disease but not during the clearance of attenuated RABV from the CNS tissues. Comparison of therapeutic immune clearance of RABV and CNS autoimmunity (EAE) indicates that BBB permeability changes can occur in the absence of neuropathology provided that cell invasion is restricted [56]. Also, it is known that the BBB permeability changes, collaboration of VNA and inflammation in the CNS plays a crucial role in the clearance of RABV from the CNS. Opening of BBB using EAE models can be exploited for the RABV clearance from the CNS by the safe passage of peripheral VNA and other immune effectors across the CNS [60].

### By using attenuated RABVs

Laboratory-attenuated RABVs have been used for developing animal vaccines and for studying rabies immunology. Many of these attenuated RABVs can spread to the CNS from the peripheral site of inoculation but can be cleared by specific immune responses provided the immune effectors must cross the BBB. Studies by Hooper et al., [86] showed that mice lacking either B and T cells or B cells alone developed a progressive disease and succumbed to infection when infected with attenuated RABV (CVS-F3). However, mice lacking either CD8+ T cells, IFN receptors, or C3 and C4 complement components had no significant differences from normal mice in the development of disease. These studies confirm that rabies VNA is an absolute requirement for the clearance of an established RABV infection. Subsequently, it has been shown by Roy et al., [57] that the induction of innate and adaptive immunity are indistinguishable between mice infected with highly lethal SHRBV and mice infected with attenuated RABV (CVS-F3). Though, CVS-F3 and SHBRV could spread to the CNS tissues from peripheral sites of inoculation but only the attenuated RABV could be cleared from the CNS, whereas the SHBRV infected mice succumb to the disease. It is found that the specific deficit in the SHBRV-infected mice, is an inability to enhance BBB permeability and to deliver the immune effectors to the CNS, indicating that the failure to open the BBB to deliver immune effectors to the CNS leads to the lethal outcome in mice [58].

### By using recombinant RABVs expressing three copies of the G

In the past, vaccine virus were attenuated and selected by conventional method of serial passaging *in vivo* and *in vitro*, but recent advancement in biotechnology allows us develop highly attenuated live recombinant RABV by manipulating its genome targeting specific genetic elements that accounts for pathogenicity and immunogenicity using reverse genetics approach [87]. Using the reverse genetic technology, it has been shown in a fixed RV strain SADB19, that the changes in the single amino acid in the glycoprotein gene at position 333Arg → Glu completely abolished the pathogenicity in immunocompetent mice after intracranial (i.c.) inoculation [88]. Furthermore, change at position at 194Asn → Ser mutation prevents the reversion to pathogenic phenotype [89]. Duplication or triplication of this mutant G gene significantly enhanced the immunogenicity of the vaccine through higher G protein expression [90] and also decreased the chances of reversion to pathogenic phenotype [91]. Though, TriGAS is shown to be more effective than conventional rabies vaccines in inducing RABV-specific immunity, as well as in promoting immune effector delivery to CNS tissues, but it is also shown to effectively protect mice as late as 4 days after infection with lethal dose of wild-type RABV [92]. Like the CVS-F3, TriGAS has been shown to enhance the BBB permeability and stimulate a robust immune response for effective clearance of RABV from the CNS [93]. Transcriptome analysis revealed that the host-pathogen responses are responsible for RABV clearance including rapid production of VNA and the induction of factors that promote the activity of immune effectors in the brain [93], thus TriGAS can also be used as a tool for rabies therapy.

### By using recombinant RABVs expressing immune stimulating agents

Studies from our laboratory indicate that recruitment and/or activation of DCs plays an important role in enhancing protective immunity against RABV infection [94–96]. Insertion of innate immunity genes like cytokine/chemokine into vaccine candidates has been reported to increase vaccine immunogenicity by recruitment and/ or activation of DCs and B cells [96–98]. It has been found that intracerebral administration of recombinant RABV expressing GM-CSF effectively protected mice as late as 5 days after infection with wild-type RABV [95]. Administration of this rRABV by peripheral routes was not as effective. Intracerebral injection of this rRABV not only resulted in the production of VNA, but also in the enhancement of BBB permeability. However, enhancement of the BBB permeability alone is not sufficient to protect mice from developing rabies since administration of a chemokine, MCP-1, enhanced the BBB permeability, but did not significantly increase the survival rate in mice infected with wt RABV, when compared to those infected with wt RABV without MCP-1 treatment [96]. On the other hand, immunization with an inactivated RABV preparation 5 days after infection with street RABV did not increase significantly the survival rate either despite the fact that VNA was produced in the periphery. Yet, administration of MCP-1 (which can transiently enhance the BBB permeability) in mice immunized with inactivated RABV significantly improved the survival rate in mice infected with street RABV [96]. Thus the combined effects of enhancement of BBB permeability and the production of VNA are required for clearance of RABV from the CNS and prevention of mice from developing rabies. Also, it has been considered that VNA produced in situ by the invading B cells at the CNS is important in clearing RABV from the CNS, rather than VNA produced in the periphery and then transported into the CNS [79].

### By intrathecal administration of RABV vaccines

It has been shown by Baer et al., in early 1970s that the intrathe-cal administration of attenuated rabies vaccine in dogs, not only induced VNA in the CSF, but also prolonged the morbidity [99]. Recently, it has been shown that the intrathecal inoculation of rabies vaccines directly into the CSF of rabbits showing neuromuscular symptoms of rabies, led to the clearance of RABV from the CNS and their survival [100]. Thus, these studies provide evidence of RBAV clearance from CNS and possible therapy for rabies using intrathecal RABV immunization.

### By administration of VNA and transient opening of BBB transiently using MCP-1

**Figure 1:**
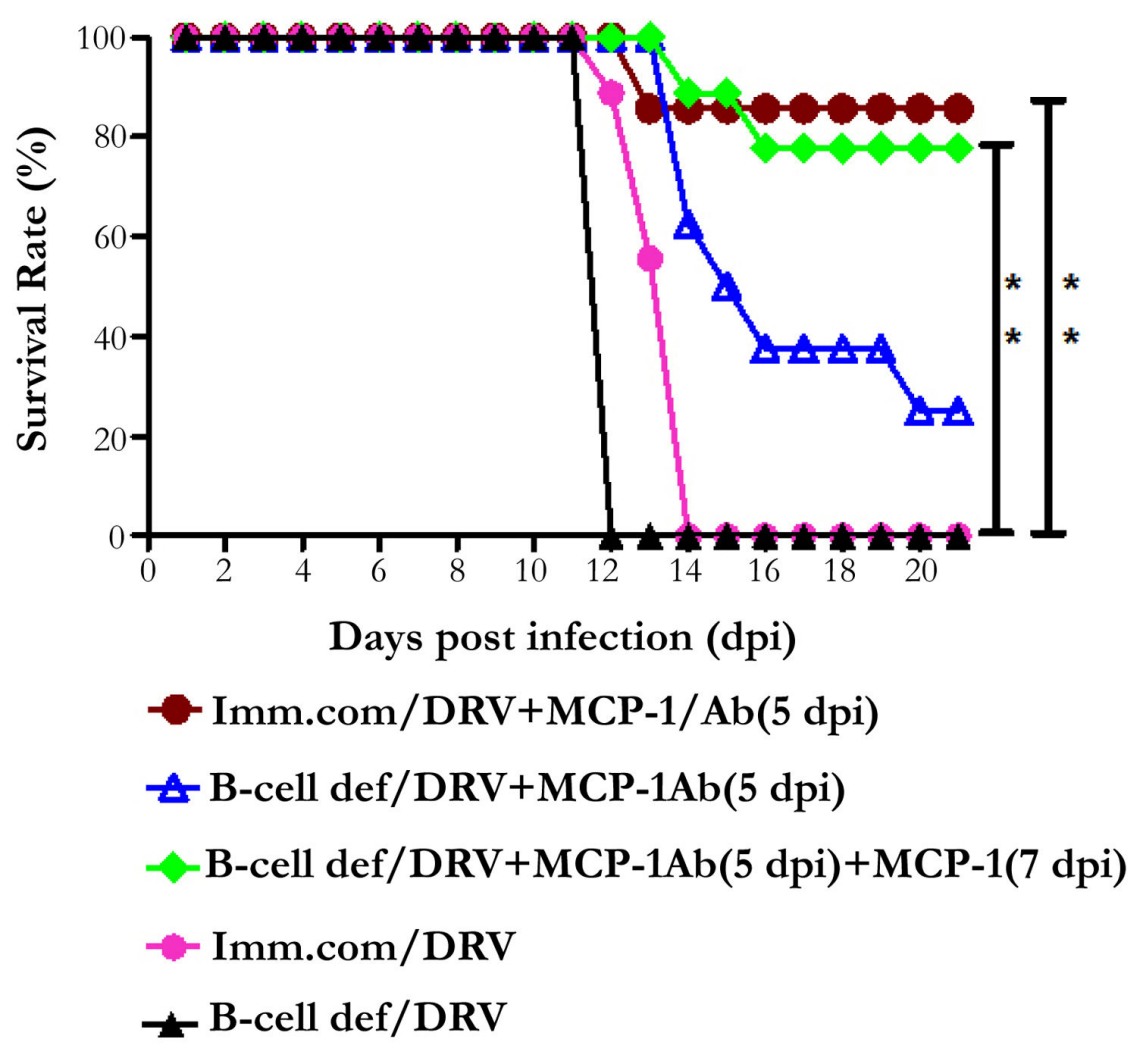
Rabies treatment by peripheral administration of VNA and MCP-1 and its protective efficacy in immunocompetent and B-cell deficient mice treated 5 days after lethal challenge. Normal and B-cell deficient mice were infected i.m with DRV and then treated with serum containing rabies-antibodies (Ab) in conjunction with MCP-1 at 5dpi or at both 5 and 7 dpi.

Although, the live-attenuated RABVs (described above) are capable of clearing rabies virus from the CNS and thus could be used as possible agent for rabies therapy, however it poses safety concerns for human use and investigation of alternative methods without safety concerns are needed. Recent studies, however, indicates that intravenous administration of VNA together with MCP-1 that can transiently enhance the BBB permeability modulating agent resulted in the clearance of RABV from the CNS and prevented the development of rabies when given 5 days post infection with wt RABV [101]. As shown in the Figure. 1, untreated, B-cell deficient or C57BL/6J (immunocompetent/ background) mice all died by 12-14 dpi. However, 80% of the immunocompetent mice treated with serum containing VNA and MCP-1 survived. It is also observed that, only 25% of the B-cell deficient mice survived the lethal rabies challenge after treatment with serum containing VNA and MCP-1 at 5 dpi despite the fact that VNA remained in high titers in the serum. Considering the transient effects of MCP-1 on BBB permeability, an additional dose of MCP-1 at 7 dpi, significantly enhanced the survival rate from 25% to 78% (Figure. 1). Thus, it is demonstrated that, VNAs administered in the periphery can clear lethal RABV from the CNS in both immunocompetent and immunocompromised mice as long as the BBB permeability remains enhanced [101]. These studies may provide the foundation for developing VNA therapy for clinical rabies.

## Animal model for rabies therapy/treatment

**Figure 2:**
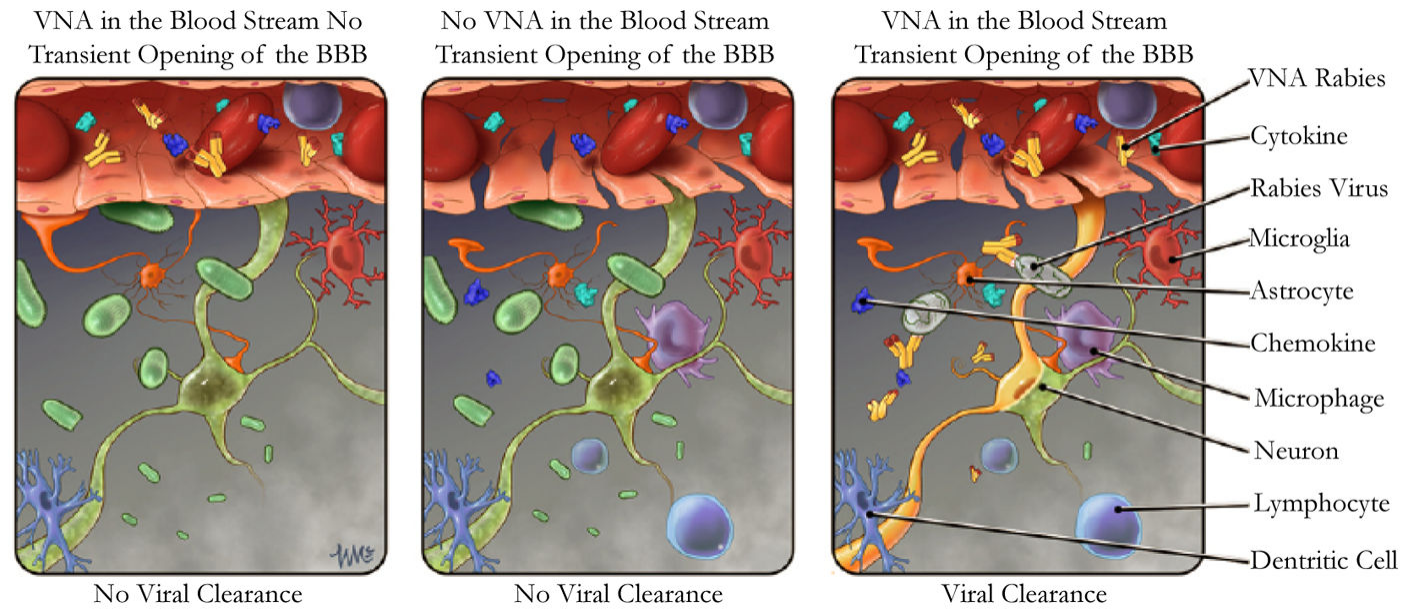
Illustration of RABV clearance from CNS that requires transient opening of the BBB, which allows the entry of immune effectors (VNA) from the periphery.

Mice have been used extensively as an animal model in deciphering rabies pathogenesis and also for the development of vaccines because of their shorter incubation and disease periods and they are available cheaply in large numbers. Although the mouse model is very useful to test the efficacy of RABV vaccines administered prior to challenge, but it is not a suitable model for developing therapy for clinical rabies because of the shorter incubation and disease periods. Dogs are one of the natural hosts for rabies and the incubation period (1–3 months) and disease process (3–7days) in dogs are very similar to those recorded in humans, thus serving the best suited animal model especially for the development of rabies therapy for humans. Our recent studies indicate that the dogs which succumbed to rabies had severe inflammation in the CNS and little or no VNA in the serum or CSF. On the other hand, the dogs which recovered rabies had high level of VNA in the CSF. Therefore it is evident that the production of VNA within the CNS or invasion of VNA from the periphery into the CNS via compromised BBB is important for clearing the virus infection from the CNS [102]. Also, studies in mice indicate that the direct administration of VNA along with the enhancement of BBB permeability can clear the RABV from the CNS. Thus, dogs would be an apt model to apply the acquired from mouse/ other studies. Our current understanding of rabies virus clearance is illustrated in Figure. 2 for effective treatment of clinical rabies.

## Future research directions for rabies treatment

### Immune clearance of RABV from CNS using bi-specific antibodies

Although drugs have been found to breach the BBB integrity, there are several issues regarding compromising the BBB, one of which is the unwanted immune molecules that enter into the CNS from the periphery may lead to neurological complications. It has been shown in experimental cerebral malaria, vascular leakage is closely associated with brain edema, coma and death [103]. The most acknowledged approach to treat rabies is to avoid meddling with BBB, by using bi-specific antibodies that can cross BBB without altering its function. It has been shown in mice, that a bi-specific antibody [one arm specific to TfR (transferrinreceptor) but rather and other arm is specific to enzyme β-secretase] can efficiently cross the BBB and reduced the accumulation of amyloid-β peptide in the CNS [104]. Therefore it is plausible to construct a bi-specific antibody, one arm recognizing the TfR, which is highly expressed by the endothelial cells that make up the BBB and other arm recognizing rabies G protein (with neutralizing ability). Thus, such bi-specific antibodies which retain the ability to cross the BBB and to neutralize RABVs in the CNS would help us to establish an effective therapy for rabies in humans.

### Neuroprotective therapy for rabies

Although, RABV clearance from CNS is the first and crucial step towards rabies treatment, however it is equally important to ameliorate the neuronal injury for complete recovery from rabies. Despite extensive investigation, the mechanism by which RABV infection causes neurological disease and damage is still not completely understood. It is shown that RABV infection causes dysfunction of ions channels, such as reduction in sodium and potassium channels [105, 106]. Also, there are evidence of impaired release of neurotransmitters such as serotonin, norepinephrine and dopamine at synaptic junctions which could result in functional impairment [3]. Investigation of structural alterations of neuronal processes in rabies infection showed severe destruction and disorganization of neuronal processes in mice infected with pathogenic but not in attenuated rabies virus. Detailed structural analysis using electron microscopy indicates loss of synaptic structures and vesicles, suggesting pathogenic RABV infection causes degeneration of neuronal processes by disrupting cytoskeletal integrity [107]. All these evidence indicate that rabies infection causes severe damages to neurons. Though, it is unclear whether the rabies induced neuronal damages are reversible but it is critical to ameliorate the neuronal injury, as a part of rabies therapy for complete recovery.

It is self-evident that the adult mammalian brain and spinal cord do not regenerate after injury or damage, but recent discoveries have forced a reconsideration of this accepted principle. As reported by Cajal in 1928 that the adult CNS neurons could regrow if they were provided access to permissive environment of a conditioned sciatic nerve [108]. Aguyao et al., replicated these studies with new methods that confirmed the regenerative capabilities of adult neurons [109]. It is clear from these findings that the failure for CNS neuron to regenerate is not an intrinsic deficit, but rather a characteristic feature of the damaged environment that did not support regeneration. In the past few years the elements promotes neurons regeneration or inhibition have been discovered. Neurotrophins (NT) which includes, nerve growth factor (NGF), brain-derived neurotrophic factor (BDNF), NT-3, NT-4/5, and NT-6, are a family of closely related proteins that were first identified as survival factors for sympathetic and sensory neurons and since have been shown to control survival, development and function of neurons in both central and peripheral nervous system [110]. Beside these, they are believed to be at least partially responsible for axon guidance and maintenance of CNS integrity [111–113]. Thus, these studies highlight the importance of NT on neuronal survival and regeneration. It would be interesting to explore the beneficial effect of “neurotrophins” in rabies therapy, by generating a chimeric attenuated RABV expressing BDNF or NGF. Such chimeric attenuated viruses could play dual role on virus clearance from CNS and also could provide a permissive environment for the rejuvenation of neurons. Such studies in animal models aimed at treating rabies using novel approaches would contribute to the establishment of an effective therapy for human rabies.

## Conclusion

The intent of this review is to provide insight into the current understanding of rabies pathogenesis for the purpose of developing and evaluating efficient treatments. Despite significant progress, rabies remains an important global disease. Successful vaccines have been developed for post-exposure prophylaxis. Yet, PEP is ineffective once clinical signs have appeared indicating active virus replication in the CNS. Recent studies aimed at improving the efficacy of PEP of rabies or clearance of virus from the CNS indicate that the presence of RABV-specific immunity (VNA) in the CNS and the transient opening of BBB permeability is an absolute requirement for effective virus clearance from the CNS. In principle, it has been shown in mice that various live-attenuated rRABVs like TriGAS or rRABVs expressing chemokine/ cytokines can induce high levels of VNA in the serum and are also capable of transiently opening BBB, resulting in the clearance of RABV from CNS and prevention of mice from developing rabies. Intravenous administration of VNA and MCP-1 (shown to transiently open up BBB) can clear lethal RABV from the CNS in both immunocompetent and immunocompromised mice, as late as 5 days after lethal challenge. However, for an effective treatment and recovery, it is not only important to clear the virus from CNS but also to ameliorate the neuronal injury induced by the viral infection. Therefore, a combination of therapy including antiviral therapy, immunotherapy and neuroprotective therapy should be an important area of research for the treatment of rabies. Such studies in animal models aimed at treating rabies using novel approaches would contribute to the establishment of an effective therapy for human rabies.

## Figures and Tables

**Table 1:** Striking difference between pathogenic and non-pathogenic RABV biology.

	Non-pathogenic virus (fixed/lab-adapted)	Pathogenic virus (street/wild-type)	References
**Cellular Tropism**	Not exclusively neuronal	Highly neuronal	(47, 114)
**Glycoprotein Expression Levels**	High	Low	(55, 114)
**Replication (titer)**	High	Low	-47
**Apoptosis**	High	Low	(114, 115)
**Interferon sensitivity**	Resistant	Highly sensitive	-98
**Immune System**	Activates innate/adaptive immunity	Evades innate/adaptive immunity	(51, 116, 117)
**Blood-brain-barrier (BBB) permeability**	Enhances	Little or no change	(53, 56–58, 79, 81, 92)
